# Message Delivery Strategy Influences Willingness to Comply With Biosecurity

**DOI:** 10.3389/fvets.2021.667265

**Published:** 2021-06-25

**Authors:** Scott C. Merrill, Luke Trinity, Eric M. Clark, Trisha R. Shrum, Christopher J. Koliba, Asim Zia, Gabriela Bucini, Timothy L. Sellnow, Deanna D. Sellnow, Julia M. Smith

**Affiliations:** ^1^Department of Plant and Soil Science, University of Vermont, Burlington, VT, United States; ^2^Gund Institute for Environment, University of Vermont, Burlington, VT, United States; ^3^Department of Computer Science, University of Victoria, Victoria, BC, Canada; ^4^Department of Community Development and Applied Economics, University of Vermont, Burlington, VT, United States; ^5^Nicholson School of Communication, University of Central Florida, Orlando, FL, United States; ^6^Department of Animal and Veterinary Sciences, University of Vermont, Burlington, VT, United States

**Keywords:** message efficacy, experimental game, compliance, numeric message, linguistic message, graphical message, risk, uncertainty

## Abstract

As the Covid-19 pandemic continues worldwide, it has become increasingly clear that effective communication of disease transmission risks associated with protective behaviors is essential, and that communication tactics are not ubiquitously and homogenously understood. Analogous to Covid-19, communicable diseases in the hog industry result in millions of animal deaths and in the United States costs hundreds of millions of dollars annually. Protective behaviors such as preventative biosecurity practices are implemented to reduce these costs. Yet even with the knowledge of the importance of biosecurity, these practices are not employed consistently. The efficacy of biosecurity practices relies on consistent implementation and is influenced by a variety of behavioral factors under the umbrella of human decision-making. Using an experimental game, we collected data to quantify how different messages that described the likelihood of a disease incursion would influence willingness to follow biosecurity practices. Here we show that graphical messages combined with linguistic phrases demarking infection risk levels are more effective for ensuring compliance with biosecurity practices, as contrasted with either simple linguistic phrases or graphical messages with numeric demarcation of risk levels. All three of these delivery methods appear to be more effective than using a simple numeric value to describe probability of infection. Situationally, we saw greater than a 3-fold increase in compliance by shifting message strategy without changing the infection risk, highlighting the importance of situational awareness and context when designing messages.

## Introduction

As the current Covid-19 pandemic sweeps across the globe, a second pandemic is raging through hogs: African swine fever is devastating swine industries, evidenced by the millions of hogs killed in Asia and Africa in 2019–2020. Endemic diseases such as Porcine Reproductive and Respiratory Syndrome (PRRS) and Porcine Epidemic Diarrhea virus (PEDV) cost over a billion dollars annually in the U.S., with PRRS alone estimated at over $600 million (1). Biosecurity, defined here as management practices designed to reduce the spread of disease, can be used preventively to reduce the likelihood of disease incidence. Preventative biosecurity generates private and public benefits. Yet, biosecurity practices come with both upfront costs, such as building a facility to clean trucks after hog transport, or opportunity costs (e.g., time required to properly sanitize boots). Waiting to develop biosecurity until the risk of a disease is imminent increases costs based on the old adage “Good, fast and cheap: Pick two.” Costs may be associated with development of biosecurity capacity, or could be associated with consistent adherence or compliance with existing biosecurity practices. In either case, one key, understudied component of biosecurity efficacy is the human component. Biosecurity is carried out by humans, both in planning and in day-to-day operations, and thus carries very real complexity and risks associated with behavior and decision-making.

Human behavior and decision-making dictate the likelihood of biosecurity lapses that can lead to disease outbreaks. Simple mistakes are difficult to completely prevent, but may be limited with training. Breaks in compliance associated with intentional decisions can be reduced using a variety of strategies such as behavioral nudges (2). Yet there are challenges to shifting behavior because factors motivating behavior are varied and complex. For example, workers at production facilities may be less willing to wash their hands for the appropriate length of time as they are leaving after a long shift. Many opportunities exist for decisions detrimental to herd health and opposing good biosecurity practices.

Human decision-making is influenced by a variety of socio-psychological factors (3, 4), including how the risk of animal infection is communicated (5, 6). Moreover, decision-making is decidedly heterogeneous and responses to the same information may differ dramatically between individuals (7).

Here, risk communication is intended to motivate changes in behavior by disseminating disease information. Within the risk communication literature, the advantages and shortcomings of different messaging styles relate to how a message is framed and presented (e.g., numeric, linguistic, and graphical or visual messages) as well as the context in which it is delivered (8–12). Numeric messages employ precision, but are likely to be poorly understood given that around 50% of our population has minimal quantitative literacy (13) and individuals with low numeracy frequently rely on numerical context (e.g., framing) to direct their behavior (14). Linguistic messaging formats can be more easily grasped in certain contexts, but lack the precision inherent in a numerical message. Graphical or visual formats have been identified as increasing salience in certain contexts, due to their ability to convey patterns and relationships (15). Despite the lack of a unifying solution, it is evident that the type of message has an effect on individual risk perception and consequent behavior (9, 10, 16).

To test how risk information may influence behavior, we created an online experimental game simulating a worker's day in a hog production facility. At one point during each day, participants are asked to exit the facility to perform a task. To exit, participants must decide to either comply with a shower-in, shower-out biosecurity practice, or leave through the emergency exit. Leaving through the emergency exit has the potential for increased earnings, but also the risk of a costly disease incursion. In essence, this choice boils down to either accepting less money by choosing the safe, biosecure option or taking a chance to get more money but with the possibility of monetary loss. This simple binary choice is influenced by the risk information provided to the participant about the chance of infection if they decide to gamble when they exit the building. Participants in this experiment are told in advance that they will make actual U.S. dollars based on their performance during the experiment. Incentive compatible, performance-based incentives such as these have been found to increase engagement and salience in experiments (15, 17).

**Figure 1:**
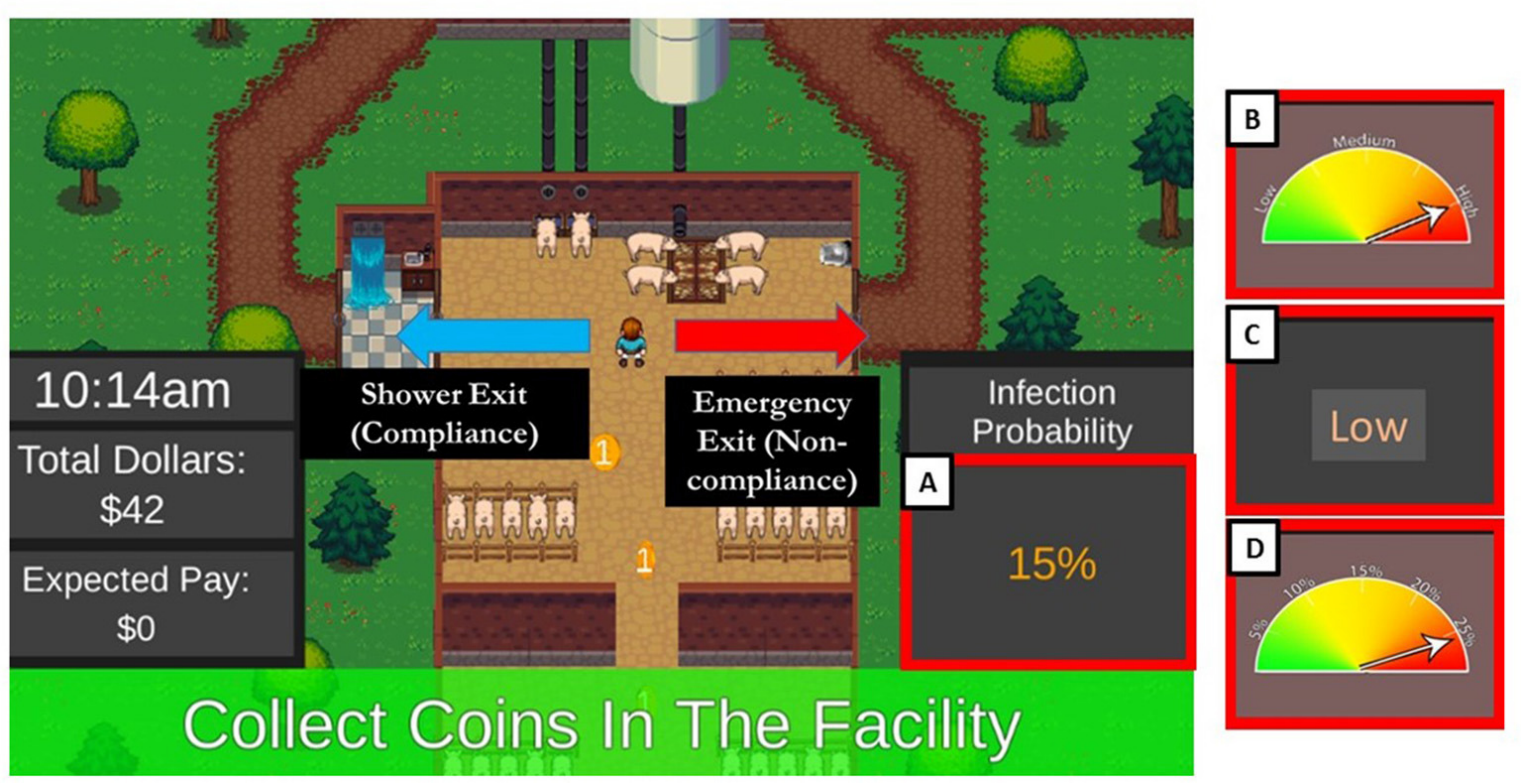
Depicted is the decision point during the experiment. This screen grab shows **(A)** the Numeric risk message format as the current treatment. Additional treatment formats used to depict risk are displayed on the right: **(B)** Linguistic Threat Gauge, **(C)** Linguistic phrase, and **(D)** Numeric Threat Gauge message format.

Here we sought to understand the influence of the format of the risk information presented to the participant about their decision to comply with the biosecurity practice. We tested four risk communication message formats: (1) Numerical, (2) Linguistic, (3) a threat gauge demarked with numeric increments (Hereafter referred to as Numerical Threat Gauge), and (4) a threat gauge demarked with linguistic increments (Hereafter referred to as the Linguistic Threat Gauge) (Figure 1). We refer to these four formats as “treatments.” Additionally, we sought to understand how depicting infection risk as a fixed estimate or value (Certain) might alter behavior, as contrasted with describing infection risk as a best estimate with a range of possible values (Uncertain).

Building off previous research (5), we hypothesized that compliance with the shower-in, shower-out biosecurity practice would progressively increase from a relatively low compliance with risk communicated using a Numerical format, then higher frequencies of compliance with the Linguistic format and the most frequent compliance observed with the two threat gauge message formats. Of the threat gauge formats, we hypothesized that a Linguistic Threat Gauge format would generate a higher frequency of compliance than information delivered using a Numeric Threat Gauge.

## Methods

### Recruitment, Experimental Design, Development, and Economics

Participants were recruited using Amazon Mechanical Turk (mTurk), an online survey recruitment platform (18). mTurk has been validated as a source for high quality data for conducting research (19). Institutional Review Board-accepted protocols were followed for an experiment using human participants (University of Vermont IRB # CHRBSS-16-232-IRB).

Data were gathered using a serious game methodology. Game design matches that used in Merrill et al. (5) but with differences in risk communication format treatments. Here, participants completed an experimental game in which they were instructed that their performance would dictate the amount of money they would earn converted from experimental dollars to real U.S. dollars at a rate of $350 to $1 U.S. Participants interacted with the simulation by using a keyboard to move their character around a hog production facility. In the beginning of each round, participants completed tasks within the facility. Once per round, a truck would arrive outside the facility, prompting a binary decision by the participant. Participants were provided information about the likelihood that their animals would become sick, thus incurring an associated cost, if they were to bypass the biosecurity practice by using the emergency exit (Figure 1). Participants would then decide to either: (1) Quickly get to the truck by avoiding the time-expensive shower biosecurity practice but risk their animals becoming sick which resulted in a loss of $50 plus potential earnings collected during the round; or (2) Choose the safe option by adhering to the shower-in, shower-out biosecurity practice, incurring the monetary costs associated with the time required to shower, but removing the risk of a disease-related loss. Given the associated costs, the choice to bypass the shower-in, shower-out biosecurity exit carried a potential benefit of approximately $9.20 experimental dollars, and a potential cost of $82.48 dollars ($50 plus the average they would have made if they used the biosecurity practice). This means that the optimal economic decision was to skip biosecurity when the risk was 1% (Very Low) or 5% (Low) but to use biosecurity when the infection risk was 15% (Medium) or 25% (High). Associated costs for each of these when skipping biosecurity are as follows: (1) when infection risk was 1% or Very Low, expected cost for skipping biosecurity was -$7.96 (i.e., the negative cost indicates that participants were likely to make money by bypassing the biosecurity practice), (2) when infection risk was 5% or Low the expected cost was -$2.99, (3) when infection risk was 15% or Medium the expected cost was $9.42, and (4) when infection risk was 25% or High the expected cost was $21.84.

Data analyzed included the dependent variable, the binary decision of whether or not to comply with biosecurity, and the independent variables associated with the infection risk information.

The compliance game platform used to administer the experiment was developed using Unity software (Unity Technologies, Version 5.3.5f1), hosted online using WebGL (20)as described in detail in Merrill et al. (5).

### Treatments

Four treatments were tested. Each treatment was designed to provide information about the risk that participants could face if they chose to exit the building without complying with the shower-in, shower-out biosecurity practice (Figure 1). The four risk information treatments were:

1. Numeric: Risk information displayed numerically: 1, 5, 15 or 25%
2. Linguistic: Risk information displayed linguistically: “Very Low,” “Low,” “Medium” or “High”
3. Numeric Threat Gauge: Risk information displayed using a threat gauge with an arrow pointing to a number: 1, 5, 15 or 25%
4. Graphical Threat Gauge: Risk information displayed using a threat gauge with an arrow pointing to a linguistic phrase: “Very Low,” “Low,” “Medium” or “High.”

### Covariates

All four treatments were implemented with four risk levels denoting the probability of infection: 1%/Very Low, 5%/Low, 15%/Medium, and 25%/High. Additionally, each treatment and infection risk level grouping was played using two levels of certainty: (1) certain risk–a single, fixed risk value, and (2) uncertain risk–an estimate with a range of risk values. This generated 32 combinations of the treatments and covariates. Because of the length of time required to complete each of these combinations in a single sitting, and concerns of experimental fatigue, we decided to have each participant play 24 of the 32 (75%) combinations, acquiring samples across all treatments using an incomplete block design.

### Analysis: Logistic Regression Mixed Effects Model

All analyses were completed using R (21). We used a mixed effects logistic regression model. The decision whether or not to use the biosecurity practice was quantified as a binary variable and was regressed against the message delivery treatment, the uncertainty covariate, the infection risk covariate as well as two-way interactions. Participant was treated as a random variable.

## Results

### Recruitment

Similar to recruiting efforts from Merrill et al. (5), we recruited 140 individuals from mTurk to participate. The experiment used four blocks, each with 35 individuals. Each participant completed 75% of the scenario set, resulting in 105 decisions for each of the 32 treatment combinations, totaling 3,360 binary compliance decisions. On average, a decision to use the shower-in, shower-out practice made $32.48 experimental dollars, whereas, a decision to skip the biosecurity practice made $41.68 experimental dollars when their animals did not become infected. If their animals became infected, they lost all accrued experimental dollars from that scenario plus an additional $50 experimental dollars. Eighteen of the 140 individuals indicated that they lived or worked on a farm or were a farmer.

### Treatments: Risk Communication Message Format

The Numeric message format had the lowest compliance with 58.1% compliance, followed by the Numeric threat gauge (67.2%) and the Linguistic phrase (67.5%). Information displayed using the Linguistic Threat Gauge resulted in the highest overall frequency of compliance (71.7%). We found some evidence for a difference between Numeric and Linguistic message formats (*p* = 0.0587, *z*-value = 1.891). Good evidence exists for differences between Numeric format and the Numeric Threat Gauge format (*p* = 0.003, *z*-value = 2.931), Numeric format and the Linguistic Threat Gauge (*p* < 0.001, *z*-value = 3.871), Linguistic message format and the Linguistic Threat Gauge (*p* = 0.037, *z*-value = 2.081). Evidence does not support other differences between treatments. High variability in decision making was observed for the Numeric Threat Gauge, indicating an inconsistent response to that message format.

### Covariates and Interactions

Unsurprisingly, infection risk level was a strong predictor of behavior with significantly increasing levels of compliance as risk increased from 1% (1% infection risk observed compliance = 26.3%. Five percent infection risk observed compliance = 54.2%, odds ratio = 9.38, *p*-values < 0.001. Fifteen percent risk observed compliance = 89.8%, odds ratio = 272.45, *p*-values < 0.001 and 25% risk observed compliance = 94.3%, odds ratio = 768.68, *p*-values < 0.001). Here odds ratios describe the odds of choosing the shower practice compared to the intercept (1% Certain Numeric message). An odds ratio of 1 (or 1/1) indicates that it was equally probable that the participant would skip or select the biosecurity practice. An odds ratio of 10 (or 10/1) indicates that the participant was 10 times more likely to choose the shower practice under those conditions. Compliance tended to increase when treatments were presented with Uncertainty (an estimate plus a range of possible values) compared to Certain estimated values of risk; Contrasted with the intercept (Certain Numeric message), messages delivered with Uncertainty resulted in the Linguistic phrase odds ratio = 1.08, *p*-value = 0.821: Numeric Threat Gauge odds ratio = 2.64, *p*-value = 0.004 and Linguistic Threat Gauge odds ratio = 2.42, *p*-value = 0.012. Uncertainty against the Certain Numeric message carried an odds ratio = 1.21 and a *p*-value = 5.39. Thus, the overall signal is that uncertainty seems to increase the willingness to forgo potential extra profits by using the shower-in, shower-out biosecurity practice. Further details regarding overall compliance with the biosecurity practice are found in Table 1 and Supplementary Table 1.

**Table 1:** Frequency of observed use of the shower-in, shower-out biosecurity practice (compliance) by treatment and covariate interaction.

**Treatment: infection** **risk message**	**Infection** **risk**	**Infection** **certainty**	**Observed** **frequency**
Numeric	1	Certainty	0.133^††^
Numeric	1	Uncertainty	0.181
Linguistic	1	Certainty	0.248
Linguistic	1	Uncertainty	0.200
Num. Threat Gauge	1	Certainty	0.238
Num. Threat Gauge	1	Uncertainty	0.391
Lin. Threat Gauge	1	Certainty	0.276
Lin. Threat Gauge	1	Uncertainty	0.438^†^
Numeric	5	Certainty	0.419
Numeric	5	Uncertainty	0.476
Linguistic	5	Certainty	0.524
Linguistic	5	Uncertainty	0.686
Num. Threat Gauge	5	Certainty	0.381^††^
Num. Threat Gauge	5	Uncertainty	0.667
Lin. Threat Gauge	5	Certainty	0.457
Lin. Threat Gauge	5	Uncertainty	0.724^†^
Numeric	15	Certainty	0.819^†^
Numeric	15	Uncertainty	0.848
Linguistic	15	Certainty	0.924
Linguistic	15	Uncertainty	0.933
Num. Threat Gauge	15	Certainty	0.848
Num. Threat Gauge	15	Uncertainty	0.905
Lin. Threat Gauge	15	Certainty	0.952^††^
Lin. Threat Gauge	15	Uncertainty	0.952^††^
Numeric	25	Certainty	0.895
Numeric	25	Uncertainty	0.876^†^
Linguistic	25	Certainty	0.943
Linguistic	25	Uncertainty	0.943
Num. Threat Gauge	25	Certainty	0.981^††^
Num. Threat Gauge	25	Uncertainty	0.971
Lin. Threat Gauge	25	Certainty	0.971
Lin. Threat Gauge	25	Uncertainty	0.962

† *Indicates lowest observed frequency per infection probability category*.

†† *Indicates highest observed frequency per infection probability category*.

## Discussion

Our contribution to the literature may be to help understand how to best communicate risk, thus increasing behavior that could reduce the spread of disease. When facing the Covid-19 pandemic it is apparent that improving the efficacy of risk communication will help save lives and reduce the impact of disease outbreaks. Our experiment, which tests message formats for delivery of disease risk information, reveals compelling insights especially with the swine production industry facing the threat of African swine fever, and our society coming to terms with a global pandemic. The results from our experiments can benefit stakeholders who seek to foster a biosecure culture in their production facilities, and may highlight communication tactics that could help broader risk communication strategy.

### Treatments: Risk Communication Message Format

**Figure 2:**
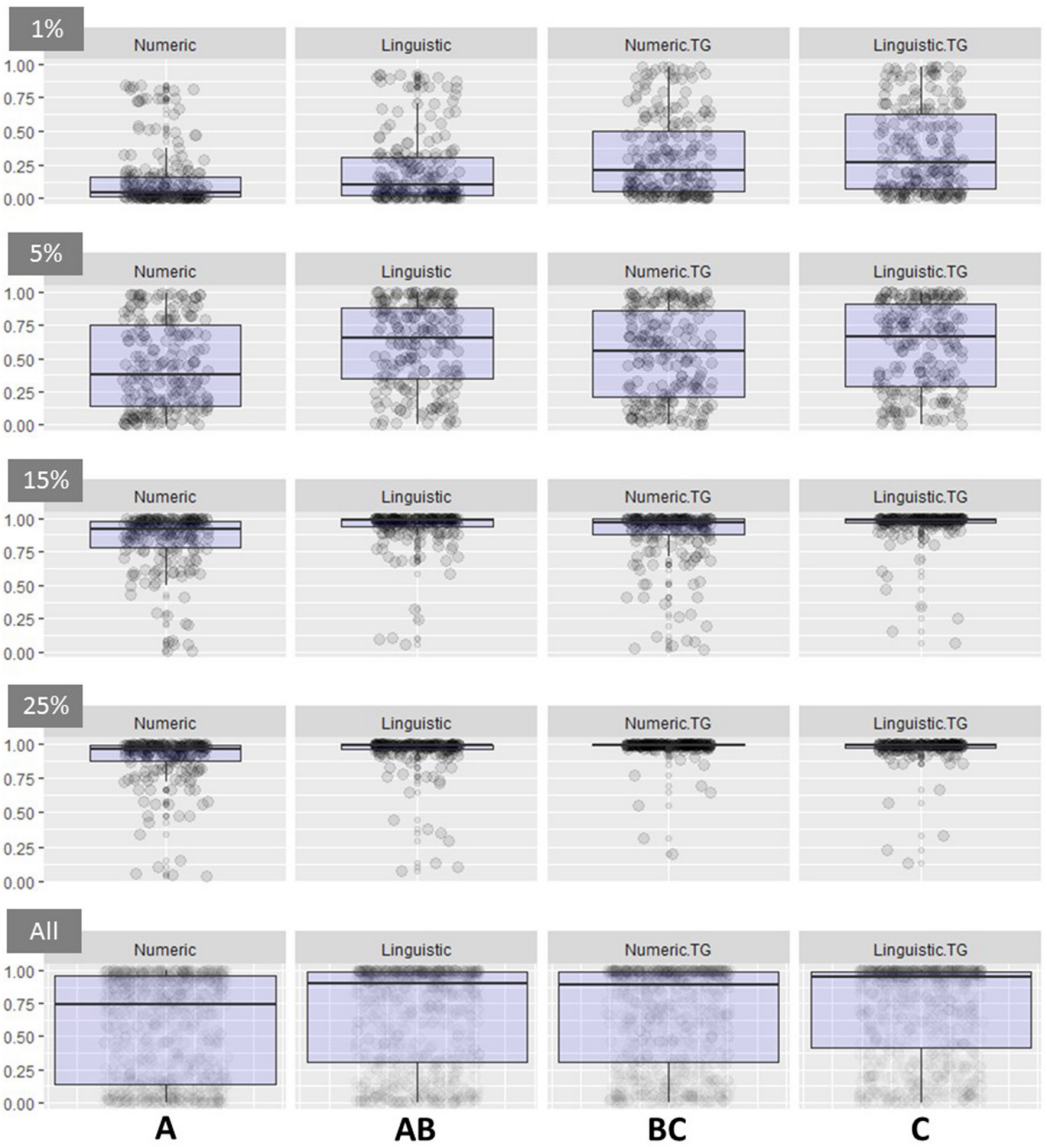
Box plot depicting results from the Mixed Effect Logistic Regression model for each of four levels of Infection Risk and the combination of all infection risk categories (Rows: 1, 5, 15, and 25%, All infection risk categories combined). The y-axis reports the probability of compliance with the biosecurity practice. Columns depict treatments (Left to Right: Numeric, Linguistic, Numeric Threat Gauge, and the Linguistic Threat Gauge. Significance between treatment categories is noted by bold letters on the bottom of the figure.

Behavioral responses to the four risk communication formats were somewhat surprising. We hypothesized that information displayed using either of the threat gauge treatments would result in increased willingness to comply with the shower-in, shower-out biosecurity practice. Merrill et al. (5) found that compliance was highest with the use of a linguistic threat gauge, over a linguistic phrase or a numeric value. Those results were replicated. However, compliance when risk was displayed using the Numeric Threat Gauge was not significantly higher than the compliance observed when risk was displayed using a Linguistic phrase. Risk communication using a Linguistic Threat Gauge was associated with the highest compliance–at ~72%–across all scenarios. In contrast, risk communicated numerically was correlated with the lowest frequency of compliance with the shower-in, shower-out biosecurity practice at approximately 58%. An intermediate level of compliance was seen with the Linguistic treatment and Numeric Threat Gauge at 67.5 and 67.3%, respectively, and were not significantly different from each other. Differences between risk communication treatments become more distinct when we look at interactions with the infection risk covariate (Figure 2) (22).

### Covariates: Infection Risk and Infection Risk Uncertainty

Supporting previous research, infection risk was confirmed as a dominant driver of decision-making strategy (5, 6). Most individuals, regardless of risk communication message format, complied with the biosecurity practice when the risk of infection was 15% with an average of 89.8% compliance and when the infection risk was 25% with an average compliance of 94.3% (Table 1). Substantial variability in compliance with biosecurity was observed in the lower risk infection categories. When risk was “low” or 5%, we observed a mean frequency value of 54.2%, ranging between 38.1% (Numeric Threat Gauge with Certainty) to 72.4% (Linguistic Threat Gauge with Uncertainty). The lowest infection risk tested was 1%, and, as expected, correlated with the lowest frequency of biosecurity compliance (mean frequency of 25.6%). Similar to the 5% infection risk category, high variability was observed when infection risk was very low or 1% with compliance frequency values ranging from 13.3% (Numeric format with Certainty) to 43.8% (Linguistic Threat Gauge with Uncertainty).

Supporting previous research (5, 6), uncertainty in the infection risk tended to increase willingness to use the biosecurity practice. This uncertainty effect was typically more pronounced at the 1 and 5% infection risks where high variability in responses was noted by treatment and covariate combination (Table 1).

Translating findings to suggested best management practice policies should proceed, but with understanding of some of the limitations. Participants were recruited using mTurk. In a similar experimental game, detailed by Clark et al. (23), biosecurity investment behavior when confronted with disease and biosecurity information was compared between a sample of mTurk participants and a cohort of industry professionals at the 2018 World Pork Expo. While Clark and others' study was analogous, it examined willingness to directly invest in biosecurity as contrasted with foregoing opportunity for gain by using biosecurity. Data from this study was not found to differ significantly between industry professionals and mTurk participants. This surprising lack of an observed difference may stem from the broad array of potential motivating factors that influence individual behavior. In other words, while there are likely differences in behavior between industry professionals and mTurk participants, teasing those differences out may be challenging, especially given the potential overlap in the communities (i.e., ~13% of mTurk participants identified as farmers, lived on a farm or worked on a farm).

Given that behavior is complex, we sought to reduce complexity by design. Here we reduced the possible motivating factors for participant decisions to a minimum, in order to observe differences in response to risk messages. Motivating factors influencing real world decisions are much more complex and nuanced. However, we suggest that there may be underlying consistencies in message interpretation that may be leveraged. Further, our research confronts only one aspect of effective message design for on-farm workers: risk information description. To design effective messaging, other aspects need to be considered, such as providing action steps (e.g., how to use the shower-in, shower-out facility) and insuring perceived relevancy of the message to the farm worker (24).

Poor biosecurity if examined cumulatively or industry-wide, can lead to widespread disease outbreaks (25). For example, the authors showed that disease outbreaks have high likelihood to turn into pandemics in a system where the producer population is largely willing to accept risk. In contrast, in risk averse populations, disease outbreaks tend to be small in magnitude and more easily suppressed. Our results demonstrate that simple changes in the communication strategy can drive substantial behavioral shifts; in one case, we increased compliance from under 40% to over 70% of participants with no change to the actual risk of infection. In a different situation, we observed over a 3-fold increase in biosecurity compliance. Such shifts could alter the state of a system from one where outbreaks were common and widespread to a system where outbreaks are quickly suppressed.

Risk communication and message efficacy under the threat of disease is at the forefront of many of our minds. To describe the threat of contracting Covid-19, the City of Los Angeles, California has recently adopted a threat gauge display that mimics our threat gauge (26). Adopting this messaging tactic over a numeric estimate of the risk likely resulted in the reduce spread of Covid-19 and fewer resultant deaths.

## Conclusion

Here we partially confirm our hypothesis that risk information delivered using a graphical message has higher efficacy for ensuring compliance with biosecurity practices, with the significant caveat that the use of numbers in risk messages, even graphically depicted messages, appears to reduce efficacy. Overall, we suggest that message formats that include numbers are likely to be relatively ineffective in communicating risk or improving biosecurity and should be used with care during message design. Moving to the use of a graphical display, instead of a numerical display, has the potential to positively nudge behavior. As noted by Bucini et al. (25), relatively small improvements in biosecurity behavior can result in substantial economic and social benefits to livestock industries. Real-world messaging strategies may substantially impact outbreak severity, which is well-worth the comparatively limited cost of implementation. In a true outbreak situation, when the threat is imminent, message design may make a difference measured not only in economic impact but in the lives of animals and people.

## Data Availability Statement

The raw data supporting the conclusions of this article will be made available by the authors, without undue reservation.

## Ethics Statement

The studies involving human participants were reviewed and approved by Institutional Review Board University of Vermont IRB # CHRBSS-16-232-IRB. Written informed consent for participation was not required for this study in accordance with the national legislation and the institutional requirements.

## Author Contributions

SM, CK, AZ, LT, JS, EC, TLS, and DS assisted with design and conceptualization of the experiment and underlying experimental game. LT, EC, and SM helped with data curation. SM worked on data analysis and Initial manuscript drafts were created. Project funding was generated with the help of JS, SM, CK, AZ, and TLS. Experiments were conducted by SM, LT, and EC. Software development was primarily led by LT and EC. Subsequent manuscript editing and retooling was completed by all authors. All authors contributed to the article and approved the submitted version.

## Conflict of Interest

The authors declare that the research was conducted in the absence of any commercial or financial relationships that could be construed as a potential conflict of interest.
