# Supplementary material for: Message Delivery Strategy Influences Willingness to Comply With Biosecurity
Source: Front Vet Sci. 2021 Jun 25;8:667265. doi: 10.3389/fvets.2021.667265 (PMC8269999; doi:10.3389/fvets.2021.667265)
Supplement: Supplementary file 1 [file Table_1.DOCX]

# Supplemental Material

##### Table S1. Summary statistics describing fixed effects from the logistic regression model.

| **Parameter** | **Odds ratio** | **LCI Odds ratio** | **UCI Odds ratio** | **Pr(>\|z\|)** | **Significance** |
| --- | --- | --- | --- | --- | --- |
| Intercept (IR Certainty, 1% Infection Risk, numeric message) | 0.048 | 0.023 | 0.101 | p<0.001 | ******* |
| IR Uncertainty | 1.212 | 0.656 | 2.240 | 0.539 |  |
| Linguistic | 2.050 | 0.974 | 4.313 | 0.059 | **.** |
| Numeric Threat Gauge | 2.991 | 1.437 | 6.221 | 0.003 | ****** |
| Linguistic Threat Gauge | 4.252 | 2.043 | 8.848 | p<0.001 | ******* |
| IR 5% | 9.376 | 4.627 | 18.998 | p<0.001 | ******* |
| IR 15% | 272.445 | 117.706 | 630.610 | p<0.001 | ******* |
| IR 25% | 768.678 | 299.603 | 1972.166 | p<0.001 | ******* |
| IR Uncertainty : linguistic message | 1.081 | 0.550 | 2.124 | 0.821 |  |
| IR Uncertainty : Numeric Threat Gauge | 2.638 | 1.356 | 5.132 | 0.004 | ****** |
| IR Uncertainty : Linguistic Threat Gauge | 2.418 | 1.218 | 4.801 | 0.012 | ***** |
| linguistic message : IR 5% | 1.419 | 0.614 | 3.277 | 0.413 |  |
| Numeric Threat Gauge : IR 5% | 0.372 | 0.163 | 0.848 | 0.019 | ***** |
| Linguistic Threat Gauge : IR 5% | 0.447 | 0.197 | 1.017 | 0.055 | **.** |
| linguistic message : IR 15% | 2.330 | 0.824 | 6.583 | 0.111 |  |
| Numeric Threat Gauge : IR 15% | 0.496 | 0.188 | 1.311 | 0.157 |  |
| Linguistic Threat Gauge : IR 15% | 1.381 | 0.443 | 4.307 | 0.578 |  |
| linguistic message : IR 25% | 1.620 | 0.518 | 5.066 | 0.407 |  |
| Numeric Threat Gauge : IR 25% | 2.474 | 0.583 | 10.505 | 0.219 |  |
| Linguistic Threat Gauge : IR 25% | 1.114 | 0.315 | 3.940 | 0.867 |  |
| IR Uncertainty : IR 5% | 2.105 | 1.196 | 3.703 | 0.010 | ****** |
| IR Uncertainty : IR 15% | 0.880 | 0.424 | 1.826 | 0.732 |  |
| IR Uncertainty : IR 25% | 0.582 | 0.241 | 1.404 | 0.229 |  |
